# Genome Instability and Somatic Mutagenesis in Autoimmune Diseases

**DOI:** 10.3390/cancers18030513

**Published:** 2026-02-04

**Authors:** Sriram Vijayraghavan, Natalie Saini

**Affiliations:** Department of Biochemistry and Molecular Biology, College of Medicine, Medical University of South Carolina, Charleston, SC 29425, USA

**Keywords:** DNA damage signaling, adaptive immune response, autoimmune disease, cancer, inflammation, cytokines, somatic mutations, mutation signatures, high-throughput sequencing

## Abstract

The adaptive immune system plays a vital role in protecting individuals against invading pathogens primarily through its ability to discern self- versus non-self-antigens. Conditions leading to the breakdown of such immune surveillance can have devastating consequences, one of them being erroneous recognition and immune response against self-antigens, resulting in autoimmunity. Autoimmune diseases (AID) are widespread and span multiple organ systems and cellular functions. Historically, the etiology of AID is multifarious and complex owing to a mix of genetic predisposition and environmental conditions. However, in recent years the study of somatic mutations has gained traction in understanding the basis of AID. Somatic mutations commonly result from elevated DNA damage and inefficient DNA repair and have been linked to cancer. Moreover, the hyper-inflammatory microenvironment is highly conducive to the accumulation of DNA damage in immune cells. Thus, understanding the mutational burden and landscape of somatic mutagenesis in the context of AID can illuminate the basis of disease development and progression. In this review, we summarize past and current research on genome instability in AID, focusing on the nexus between inflammation, immune response, DNA damage, and mutagenesis, and discuss the possible link between AID and cancer development. We provide examples of autoimmune disorders that have been studied from a mutational standpoint and outline results from key studies highlighting the extent of DNA damage and mutagenesis in cells from AID patients. Lastly, we provide our perspective on the key challenges and future directions to understand the role of somatic mutagenesis in autoimmunity and cancer.

## Simple Summary

Autoimmune disorders (AID) are complex, multifactorial, and pervasive human health conditions that affect people globally. Nearly all AID are characterized by heightened inflammation and a breakdown of immune regulation, resulting in a loss of tissue homeostasis. Additionally, the hyper-inflammatory environment observed in AID is ideal for triggering neoplasia, resulting in various forms of cancer and further complicating disease treatment. Within the dysregulated immune microenvironment underlying AID, there is abundant DNA damage and genome instability. We posit that recurrent cycles of inflammation and DNA damage can result in stochastically elevated levels of somatic mutations in autoimmune disorders, and contribute to downstream malignancies. Understanding AID-associated DNA damage and mutagenicity would illuminate mechanisms that would subsequently inform disease prognosis, pathology, and progression. We explore this theme in our review by highlighting studies linking AID with genome instability and mutations, and carcinogenesis.

## 1. Introduction

Individuals accumulate somatic mutations through their lifetime. Typically, somatic mutations exhibit an age-associated increase in normal cells, across the spectrum of diverse cell types spanning multiple organ systems [1]. The average number of mutations accumulating per cell per year range from ~2.5 for cell types such as spermatogonia [2], to >50 in intestinal crypt cells [3]. Primarily, somatic mutagenesis is the net outcome of an interplay between DNA damage and DNA repair throughout the cell cycle [4]. The estimated number of abasic sites generated via depurination amount to roughly 10,000 per day in human cells [5]. Similarly, cytosine methylation and deamination, resulting from metabolic processes and exogenous exposures, as well as UV-associated thymidine dimerization, represent the most common ongoing pyrimidine base damages. As individuals age, cells undergo constant metabolic and cytogenetic fluctuations. This could manifest as mitochondrial dysfunction, leading to increased reactive oxygen and nitrogen species (RONS), replicative senescence, or increased autophagy [6]. Additionally, cells can undergo elevated stress signaling in response to exogenous stressors such as cigarette smoke, alcohol, occupational hazards, and pharmaceutical interventions. Under such circumstances, cells that additionally have lowered damage repair fidelity rapidly acquire mutations within their genomes. Recent studies have demonstrated that with age, the rate of accumulation of somatic mutations exponentially increases in the form of single base substitutions, insertions-deletions (INDELs), and copy number changes. Additionally, retrotransposon elements such as L1 are drastically upregulated in cells undergoing aging, as well as in age-related diseases (reviewed in [7]). While mutations in genes critical for survival are often deleterious and quickly lost from populations, many mutations, such as those occurring in tumor suppressor genes, confer selective growth advantages on such cells, allowing them to escape cellular and immune checkpoints. Unsurprisingly, widespread somatic mutagenesis is a hallmark of tumorigenesis [8], with more than half of the somatic genome changes identified in cancers found to originate from pre-malignant tissues [9].

Autoimmunity represents the breakdown of immune tolerance mechanisms that prevent B- and T-lymphocytes from directing their antimicrobial functions toward self-antigens in the host. Environmental factors, genetic predisposition, and repeated illnesses, can exacerbate lymphocyte proliferation and promote diversification of membrane antigen receptors. In the absence of regulation, enhanced activity of the lymphoid system can have pathogenic consequences, ranging from elevated autoimmune responses to cancers such as lymphoblastic leukemias. As we illustrate in the following sections of the review, autoimmune disorders and chronic inflammation are often associated with elevated rates of DNA damage, mutagenesis, and carcinogenesis. Therefore, inflammation and DNA damage make overlapping contributions to the emergence of autoimmunity and perhaps, subsequent progression to cancer.

### Primary Considerations–The Autoimmune Response

Immune responses involve pathways that coordinate with antigen receptor activation to co-stimulate lymphocyte growth. Lymphocytes lacking co-stimulation are subsequently targeted for destruction to minimize the autoimmune response. This can occur via Fas cell surface death receptor (FAS)-associated cell death in self-reactive B- and T-lymphocytes [10], suppression of co-stimulation of additional ligands such as CD28 and IL-2 in T-lymphocytes [11,12,13], overriding B-lymphocyte growth mechanisms via activation of BIM-induced apoptosis [14], and inhibition of CD40 and IL-4 receptors on B-cells [15].

Regulatory T-cells (Tregs) show depleted function in AID like systemic sclerosis (SSc), systemic lupus erythematosus (SLE), and rheumatoid arthritis (RA), among others [16,17,18]. Within tumor microenvironments, high levels of Tregs expressing inflammatory cytokine IL-10 and TGF-β, as well as the transcriptional regulator FoxP3, correlate with poor prognosis for multiple cancer types including breast, lung, and pancreas [19]. Paradoxically, FoxP3+ Tregs correlate with better survival outcomes for other cancer types such as colorectal cancer (CRC), esophageal carcinoma, and head and neck cancers [19]. Because Tregs are crucial in suppressing autoimmune response, selectively targeting tumor-associated Tregs in synergy with other cancer immunotherapies, such as PD-L1 inhibition, is an area of active research [20].

Immune checkpoint mediators such as the receptor CTLA-4 (cytotoxic T-lymphocyte antigen 4) and PD-1/PD-L1 (programmed cell death receptor/ligand) also play dual roles in suppressing autoimmunity, while promoting tumor-cell recognition and destruction. While *CTLA4* mutations are associated with almost all autoimmune disorders [21,22], loss of CTLA-4 increases the immune response to cancer [23]. Interaction of PD-1 with its ligand PDL-1 triggers destruction of auto-reactive T-cells in various tissues such as pancreas islet cells and the vascular endothelium to suppress autoimmune responses [24,25,26]. In cancer cells, persistent PD-1 and PD-L1 upregulation results in exhaustion of both CD4+ and CD8+ T-cells, and cancer progression via immune escape [27,28].

Within B-lymphocytes, mutations associated with the *PTPN22* gene that encodes the Lyp protein tyrosine phosphatase are associated with defective macrophage function, development of Tregs, and mast cell activation [29]. *PTPN22* mutations increase the proportions of self-reactive B- and T-cells, and are consequently associated with several AID [30,31,32,33]. Surprisingly, *PTPN22* variants have also been associated with lower incidence of cancer and/or better response to anti-tumor therapies [34], indicating the dual role of immune signaling in promoting immune tolerance in the host while simultaneously activating T-cell responses toward cancers.

Fas Ligand (FasL), which promotes cell death of self-reactive B-cells and T-cells [35,36], is also implicated in cancer. Excessive secretion of FasL by myeloid-derived suppressor cells (MDSCs) leads to the depletion of tumor-infiltrating lymphocytes (TILs) and results in poor cancer immunotherapy outcomes [37]. MDSCs participate in tumorigenesis via multiple mechanisms including vasculogenesis, T-cell apoptosis, epithelial-to-mesenchymal transition (EMT), and via recruiting Tregs to the tumor microenvironment [38,39,40]. Elevated MDSC populations negatively correlate with the metabolic profiles of several autoimmune disorders including RA, SLE, and T1D [41,42,43]. In experiments with *fas*-mutant mice, progression to autoimmunity is marked by a rise in the number of RNA- and DNA-autoantibodies, and reduced function of the Toll-like receptors TLR7 and TLR9 that are typically produced during anti-microbial responses [44,45]. A *FAS*-inactivating mutation in T-cell lineages combinatorially exacerbates the autoimmune phenotype by increasing the proportion of CD4+ T-cells co-expressing the CD40 ligand and the CD28 receptor [46,47].

## 2. Linking Autoimmunity and Cancer via Genome Instability

DNA damage and somatic mutagenesis are likely to be key events for the onset and propagation of autoimmunity. Goodnow et al. (2007) [48] propose in their review that acquisition of somatic mutations might be the key event for the bypass of immune checkpoints to activate an AID phenotype. Lymphocytes naturally undergo extensive somatic recombination to produce a diverse array of antibodies, in a process termed class switching recombination (CSR),an inherently error-prone process. Interestingly, somatic *FAS* mutations in a single hematopoietic stem cell can give rise to mutant B-cell populations that clinically manifest as autoimmune disorders mimicking germline FAS defects [49]. Further, genome instability, immune dysfunction, and inflammation have been proposed as key enablers of tumorigenesis [8]. As such, diseases that are immune-related and pro-inflammatory often proceed to cancer development. Understanding the underlying signaling networks, as well as the key regulators in this process, is not only crucial to determine the molecular basis of complex immune-associated pathologies, but also help determine why inflammation often begets cancer.

### 2.1. Inflammation as a Source of Oxidative DNA Damage

A possible mechanism linking DNA damage and autoimmune response is chronic inflammation. Inflammation is a potent inducer of reactive oxygen and nitrogen species (RONS), which are produced in copious amounts by immune cells, and via cytokine signaling (reviewed in [50]). RONS are abundantly generated within macrophages at sites of inflammation, wherein they drive positive feedback loops of inflammation, contribute to auto-antigen development, and therefore promote autoimmunity. To minimize RONS-associated damage, cells utilize an oxidative stress sensing pathway involving Nrf2, which is usually sequestered in the cytoplasm by Keap1 in the absence of stress, but localizes to the nucleus and turns on antioxidant response genes, including NQO1, GST, and SOD1 (reviewed in [51]). Abrogating Nrf2 has been associated with increased DNA damage and lowered repair efficiency [52,53]. Lastly, because, persistent RONS is genotoxic and mutagenic, DNA damage resulting from chronic inflammation can lead to acquisition of mutations in genes related to tumor suppression and immune regulation, and drive AID progression.

**Figure 1:**
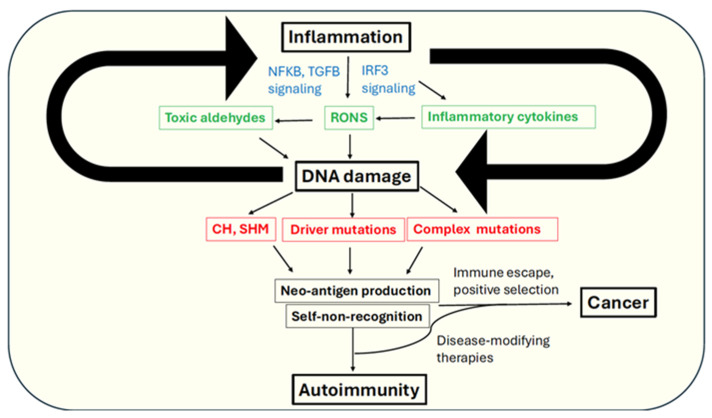
Mechanistic links between autoimmunity and cancer via inflammation and genome instability (refer text for details). RONS-reactive oxygen and nitrogen species, SHM-somatic hypermutation, CH-clonal hematopoiesis. Main effectors of inflammatory response are indicated in green. Key outcomes of DNA damage are indicated in red. Key signaling pathways are indicated in blue.

Oxidative stress can manifest in multiple forms of DNA damage. The most common DNA lesion produced via oxidative base damage is 8-oxo-guanine, which can mutagenically produce G → T transversions [54,55,56]. Oxidative deamination of cytosines or methylcytosines can also generate C → T transitions, which are ubiquitous mutations in all cancer types [57]. RONS can react with fatty acid molecules and generate a variety of toxic aldehydes, such as malondialdehyde (MDA), crotonaldehyde, methylglyoxal, and 4-hydroxynonenal (4-HNE), which can adduct guanine bases, driving mutagenesis, and replication fork stalling and double strand breaks (reviewed in [58]). Importantly, reactive aldehydes generated in this manner can not only produce neo-antigens for self-immune targeting but also modify immune tolerance against native targets, further amplifying the autoimmune response (Figure 1) [59]. Notably, aldehydes such as 4-HNE and methylglyoxal can covalently modify multiple biomolecules including proteins and DNA, with several recent studies highlighting the pervasive mutagenicity of aldehydes [60,61,62,63,64,65,66,67]. Studies have observed that antigens generated by such aldehydes on different biomolecules can molecularly “mimic” antigenic epitopes, resulting in cross-reactivity of antibodies, which has since been proposed as a potential mechanism for autoimmunity [68].

### 2.2. DNA Repair Pathways and Immune Signaling in Autoimmunity

Multiple DNA repair pathways are dysregulated in autoimmunity. The enzyme PARP1 (poly(ADP-ribose) polymerase 1) plays a key role in DNA repair, whereby it senses single strand DNA breaks (SSBs), and helps recruit the base-excision repair (BER) machinery to the damage site [69]. PARP1 activity has been directly linked to inflammation, as PARP1 inhibition results in the reduction of inflammatory cytokines, lowered immune cell infiltration, and lowered activity of inflammation-associated enzymes [70,71]. Mutations in the DNA mismatch repair (MMR) pathway genes such as *MLH1* are a common risk factor for the development of colon cancer in patients of inflammatory bowel disease (IBD) [72]. Autoantibodies to several DNA repair factors are elevated in several rheumatic diseases including SLE, SSc, and rheumatoid arthritis (RA); these include the non-homologous end joining (NHEJ) factor Ku, and DNA repair factors in the homology directed repair (HDR) pathway, such as MRE11 and PARP1 [73].

Recent studies have similarly illuminated the extensive genome instability and mutational burden underlying SSc, wherein a prominent signature of POLH-associated mutagenesis is observed, suggesting a role for error-prone translesion synthesis (TLS) pathways in AID (detailed in the Section 3, [74]). In addition, the presence of extrachromosomal DNA, for example, via leakage of nuclear DNA into the cytoplasm, can signal the innate immune response, with the loss of regulated programmed cell death (PCD) contributing to autoimmunity [75]. Additionally, cytoplasmic R-loops that result from aberrant processing of RNA: DNA hybrids, formed during transcription and replication, can trigger the immune response [76]. Using immunoprecipitation combined with whole genome sequencing, researchers showed that a subset of nuclear R-loops generated these cytoplasmic hybrids, which activated the immune sensors cGas and TLR3, increased IRF3 phosphorylation, and induced apoptosis [76]. Finally, the cGas pathway is a potent modulator of immune response via an IFNII-TRF3-autuphagy axis, which clears cytoplasmic DNA and prevents chronic immune activation and autoimmunity [77].

In a clinical study of children with autoimmune disorders in a Serbian population, thyroid diseases such as Graves’ disease and Hashimoto’s disease, and type I diabetes mellitus (T1D), patients had elevated micronuclei formation and DNA breaks in peripheral blood cells, suggesting an inter-connection between genome instability and immune response in AID pathogenesis [78]. Mutations in the transcriptional regulator of the autoimmunity gene *AIRE* prevent the destruction of their host thymus medullary epithelial cells, which subsequently enter blood circulation and trigger autoimmune responses [79].

AIRE expression is also linked to sex-specific cancers, owing to its regulation via sex hormones, with *AIRE^+/+^* prostate cancer cells secreting elevated levels of IL-6 and prostaglandin 2 (PGE2), which contribute to chemo-resistance in these tumors [80]. Further, B-lymphocytes can secrete auto-antibodies to several DNA damage-associated proteins including Ku, PARP, MLH1, and P53 in autoimmune rheumatic disease (SARD) [81].

Overall, these studies show that the loss of immune regulation can result in extensive DNA damage, which can subsequently elevate global mutagenesis and drive cancer.

## 3. Autoimmune Disorders with Elevated DNA Damage, Mutagenesis, and Cancer Risk

The intersection of DNA damage signaling and immune responses underlies the pathology of a variety of AID. As a corollary, elevated DNA damage and mutagenesis point to a molecular basis for tumor development, potentially unveiling the mechanism(s) underlying carcinogenesis in autoimmune patients (Figure 1). In this section, we individually describe a subset of autoimmune disorders that have a well-documented scientific history of genomic instability, along with elevated cancer risk. The main diseases are reviewed below, along with a summary of their clinical features and cancer risk (Table 1), and associated DNA damage and mutagenesis (Table 2).

### 3.1. Systemic Sclerosis

Systemic sclerosis (SSc, often interchangeably termed scleroderma) is an AID associated with excessive collagen production, resulting in multisystem dysfunction that results in one of the highest mortality rates among fibrotic diseases [122,123]. The predominant cause of death in SSc patients is interstitial lung disease (ILD) and pulmonary fibrosis [124], even though multiple organ systems are susceptible in SSc due to pathological vascular damage and inflammation [125]. A hallmark of SSc is an altered immune response via generation of autoantibodies to nuclear proteins, and in conjunction with inflammation, leads to increased production of TGF-β, cytokines IL-1 and IL-6, and platelet-derived growth factor (PDGF) [126]. Together, these factors propagate a cycle of fibrosis, vascular and tissue attrition, and autoimmunity [127].

The genetic determinants of SSc are confounding [128,129,130]. Studies have noted both global methylation changes, as well as differential promoter methylation across various genes in SSc-associated fibroblasts, endothelial cells, and immune cells, including Type I interferon-encoding genes, PARP1, Krüppel-like factor 5 (KLF5), and bone morphogenic protein receptor II (BMPRII) (reviewed in [131]). Similarly, both global [132,133] and local [134,135,136] epigenetic changes have been observed in SSc patients. Even so, the pathophysiology of SSc remains a complex and poorly resolved phenomenon.

SSc patients are at an increased risk of developing cancer, in particular lung cancer, compared to their age- and sex-matched cohorts. However, other cancer risk incidences are also elevated in SSc patients compared to matched normal peers, including gynecological cancers, skin cancers, and hematological cancers [83]. The timing of cancer onset in SSc patients has not been rigorously analyzed, although cancer risk is highest within the first five years of SSc disease manifestation. A survey of patients enrolled in the Australian Scleroderma Cohort Study (ASCS) showed a remarkable correlation between the appearance of autoantibodies to RNA polymerase III (RNAPIII), and the onset of cancer in patients that were recently diagnosed with SSc, perhaps suggesting elevated genome instability [137]. Conversely, it has been proposed that that heightened immune response to tumor development in precancerous cells contributes to an autoimmune phenotype that later manifests as SSc [138].

#### DNA Damage and Mutations in SSc—Link to Carcinogenesis?

Numerous studies have demonstrated genome instability in SSc patient-derived tissues. In addition to the characteristic autoantibodies to nuclear proteins [82], DNA from peripheral blood mononucleocytes (PBMCs) of SSc patients had increased SSBs and double strand breaks (DSBs) [104]. In the same study, the authors showed that treatment of SSc-derived cells with the DNA alkylating drug melphalan resulted in longer persistence of the DSB marker γH2AX compared to control cells, suggesting impaired DNA damage response in these cells. In another study, telomere attrition was observed to be much more severe in DNA from blood leukocytes from SSc samples compared to healthy controls, wherein telomere shortening additionally correlated with increasing age of patients [103]. In urine samples from SSc patients, high levels of 8-oxoG were observed compared to age- and sex-matched healthy controls, indicating underlying oxidative stress in these samples [102]. In skin fibroblasts from healthy and SSc samples, researchers found considerable centromere size variation, karyotypic abnormalities, and micronuclei-associated activation of the cGAS-STING/IFN-β pathway, providing evidence of global genome instability and inflammation [101]. Interestingly, peripheral blood cells derived from the same patients did not display the genomic defects associated with skin fibroblasts, strongly suggesting that genome instability is not a genetic feature of SSc per se, rather a somatic occurrence [101]. In a mouse model of SSc, whereby fibrosis is induced via bleomycin treatment [139], researchers observed a similar centromere instability phenotype, involving double strand breaks in the alpha-satellite centromere that are repaired via homologous recombination [140,141].

If there is prevalent DNA damage in SSc, can such damage/mutagenesis be detected? If so, genomes from SSc patients are likely to bear characteristic mutational signatures that could inform the processes that caused and/or were a consequence of the damage. Recently, Gniadecki and colleagues [105] analyzed skin biopsies from eight SSc patients and performed whole-exome sequencing to identify somatic mutations associated with the samples. They observed ~7000 combined (synonymous and non-synonymous) mutations across samples, and a diffuse mutation signature (SBS40) resembling the clock-like signature (SBS5), perhaps suggesting age-related mutational accumulation in their sample. Further, sequencing identified mutations in common cancer-associated driver genes including *KRAS*, *TP53*, and *PIK3CA*, and mutations in genes associated with genome stability, including DNA methylation (*DNMTs*), and DNA repair (*BRCA1*, *RIF1*, *WRN1*, etc.) [105].

In their elegant study, Vijayraghavan and colleagues [74] performed whole genome sequencing of single fibroblasts derived from lung explants of five healthy individuals and six SSc patients. Subsequently, they measured somatic mutation burdens across the entire genome in each single cell lineage using DNA isolated from single cell-derived clonal expansions of normal and SSc-derived fibroblasts. On average, SSc-derived genomes carried more than twice the mutational burden of control genomes, as well as a higher proportion of single base substitutions (SBS) type mutations per year than non-SSc genomes (median SBS number per year for SSc = 28.74, healthy = 12.84). Mutations appeared uniformly distributed across SSc genomes, with no apparent association with genomic features like replication or transcriptional-strand specificity [74]. In addition to single base changes, SSc samples carried an enrichment of several complex mutation types including copy number variations (CNVs), chromosomal structural variations including inversions and large-scale deletions (2–100 kb), and small (1–2 bp) INDELs.

SSc genomes bear discrete mutational patterns. A mutational spectrum analysis of the SBS mutations identified an enrichment of the COSMIC SBS signature 93, which consists primarily of mutations within the nYw trinucleotide motif (n = any base, Y = C or T, w = A or T) (https://cancer.sanger.ac.uk/signatures/sbs/, accessed on 7 December 2025) [74]. Subsequently, a parallel mutational signature deconvolution analysis revealed an enrichment of nTw → N mutations in SSc samples, which is a signature of *POLH* activity, which encodes the translesion synthesis DNA polymerase Polη (Pol Eta) [142]. Analysis of lung samples from patients of chronic obstructive pulmonary disease (COPD) also showed enrichment in this mutation signature, as well as samples from smokers/ex-smokers [74]. There was a remarkable overlap between SBS93 and the nCw → N and nTw → N mutational signature analyses, and a further enrichment of the same signature in SSc samples within mutational “clusters”. The latter represent localized genomic regions of high mutation density [106,107], and are a characteristic feature of aberrant Polη activity. These observations led the authors to conclude that the previously unknown etiology of SBS93 is likely based upon elevated Polη activity, perhaps arising as a mechanism to bypass inflammation-induced DNA damage in SSc samples. Surprisingly, a subset of SSc samples carried an enrichment of C → T base changes within a wrC motif (w = A/T, R = A or G, T is the mutated base), which is a signature of AICDA (activation-induced cytidine deaminase) activity–an immune response gene primarily active in B-lymphocytes [108]. Atypical AICDA activity has been seen in cancer genomes in non-B-lymphocytic cell types [109], raising the possibility that that chronic inflammation can trigger AICDA expression even in non-immune cells in SSc. Whether such promiscuous AICDA activity operates in other autoimmune disorders remains unexplored.

At least one SSc sample demonstrated evidence of the APOBEC (apolipoprotein B mRNA editing enzyme, catalytic polypeptide)-associated mutagenesis, and clustered mutations. APOBEC includes several enzymes in the cytidine deaminase family that primarily function in innate immunity, but often generate large clusters of mutations in a sequence-specific manner in cancer genomes [106,110,111]. The authors also noted copy number changes and structural variants across SSc genomes that were not as prevalent in normal cells. Finally, analysis of the somatic mutations demonstrated mutagenesis in genes associated with cancer initiation (*NF1*, *SEC31A*), inflammation and immune response (*CTNNA3*, *BCOR*), and DNA damage response (*CGAS*). Overall, the study offered novel insights into the multiple mutational processes operating within SSc genomes that could potentially play a key role in disease progression, as well as cancer risk [74].

Lastly, using a cohort of non-small cell lung cancer samples with systemic sclerosis, a new study identified recurrent *TP53* mutations, and an enrichment for APOBEC-associated mutation signatures SBS2 and SBS13 in patients that had both SSc and cancer [112]. In combination with prior studies, a mutational model of SSc is emerging, whereby inflammation and genome instability concurrently drive autoimmunity and favor tumorigenesis.

### 3.2. Inflammatory Bowel Diseases

The gastrointestinal tract (GI) can become chronically inflamed and ulcerated in a cluster of inter-related autoimmune phenomena collectively termed inflammatory bowel disease (IBD). IBD is globally prevalent, includes the well-known syndromes Crohn’s disease and ulcerative colitis (UC) [113]. IBD usually peaks between the ages of 20–40 while early and very early onset IBD can be seen at 6–17 years of age [114]. IBD has a confounding, multifarious pathophysiology spanning genetic predisposition, environmental exposures, gut mucosal barrier breakdown, and altered immune response [115,116]. Patients additionally have a severely dysregulated gut microbiome; the reduced proliferation of beneficial bacteria such as *Faecalibacterium* sp. and *Bifidobacterium* sp., and increased colonization of pro-inflammatory species such as *Escherichia coli* and *Enterococcus faecalis*, contribute to the overall breakdown of the intestinal barrier and attenuation of immune tolerance [117]. Patients of IBD clinically present with severe ulceration, diarrhea, rectal bleeding, and weight loss, with treatment options for moderate-to-severe UC and Crohn’s ranging from pharmacotherapies to immune modulation and surgical resection [118,119]. Although more than 200 genetic loci are implicated in the development of IBD [120], reliable diagnostic biomarkers of clinically predictive value for IBD are still lacking, with an outsized reliance on continuous monitoring of patients via invasive procedures such as endoscopy [121].

The immunogenicity underlying IBDs has been linked to genome instability. This includes the presence of extrachromosomal DNA (ecDNA), including nuclear and mitochondrial DNA, which could trigger the innate immune response via Toll-like receptors (TLRs) and result in the secretion of inflammatory cytokines. An abundance of plasma and colon ecDNA was observed with increasing intestinal inflammation in a murine colitis model [143], as well as in Crohn’s and UC patient samples [144]. Importantly, the primary risk of mortality in IBD patients is from the development of colorectal cancer (CRC) [85,86].

Several studies have analyzed the DNA damage underlying IBD. Early studies observed considerable microsatellite instability and proto-oncogenic activation in UC-associated dysplasia, and identified a role for mismatch repair factors MSH2, MLH1, and PMS2 in impaired genome stability in patients that went on to develop hereditary non-polyposis colorectal cancer (HNPCC) [72,145,146]. Lymphocytes from IBD patients had elevated levels of micronuclei formation and nucleoplasmic bridges [147], and had a high burden of oxidative DNA damage, which are likely driven by chronic inflammation, age, DNA repair deficiencies, and perhaps treatment interventions [148]. In a mouse model of colitis, whereby animals are fed dextran sodium sulfate (DSS) in drinking water, mice lacking a base excision repair glycosylase gene had higher levels of colon hyperplasia, replication fork damage, and accumulated base lesions [149]. Similar results were observed in mice lacking the MBD4 DNA glycosylase [150].

Because somatic mutagenesis is a well-established predictor for cancer development, several newer studies have focused on identifying mutational patterns that could delineate the mechanisms driving cancer development in IBD patients. Recurrent somatic mutations were observed in IBD patients with colorectal cancer, including genes in the Rho and Rac GTPase pathway, as well as in genes encoding the ERBB ligand NRG1 and cytokine IL-16 [151]. Tumors from IBD vs. non-IBD patients differed in their mutational spectra vis-á-vis *TP53* mutations [151], as well as *APC* and *IDH1* mutations [152]. In comparing colonic crypts from 46 IBD patients with varying disease severity, Olafsson and colleagues [153] leveraged whole genome sequencing to identify IBD-specific mutations. Compared to normal colonic epithelia, cells from IBD patients had almost double the SBS mutation burden and significantly elevated INDEL accumulation per year per crypt [153]. Furthermore, a variety of mutational signatures including polymerase slippage (ID1 and 2), reactive oxygen species (SBS18), and aging (SBS1 and 5) were observed in both IBD and non-cells, highlighting rampant proliferation and metabolic activity in the colonic milieu. In a subset of crypts with high mutational burdens, there was an enrichment of APOBEC signatures SBS2 and SBS13, as well as clonal expansion and selection for oncogenic drivers such as *KRAS*, *TP53*, *BRAF*, *ATM*, and *SOX9*. Similar studies using colon biopsies [154] or colonic organoids [155] utilized whole exome sequencing (WES) to identify positive selection for mutations that inactivate anti-inflammatory genes such as *NFKBIZ* and *TRAF3IP2* in non-cancer UC patients, but negative selection for the same genes in patients that develop CRC. These data suggest somewhat perplexingly that inflammation and cancer could be driven by opposing mechanisms.

A recent study analyzed somatic mutational datasets from whole-genome and whole-exome-sequenced DNA from non-cancer diseased patients, within which the colons of IBD patients (Crohn’s disease and UC) contained an enrichment of epoxide-associated aTn → aCn mutations, acetaldehyde-associated gCn → gAn mutations, APOBEC-associated tCw → tGw (w = A/T) mutations, and a general increase in age-associated (“clock-like”) mutation signatures within the nCg trinucleotide motif likely resulting from spontaneous deamination of methylated cytosines [156].

Lastly, colons of IBD patients are enriched in pathogenic variants of the Gram-negative bacterium *Escherichia coli* (*E.coli pks^+^*), which secrete colibactin–a potent genotoxin capable of inducing DNA crosslinks and double strand breaks [157,158,159]. The genotoxicity of colibactin is thought to be a key driver of CRC; mutational patterns associated with colibactin have been identified, with an enrichment in T → N substitutions in aTa, aTt, tTt contexts [160,161,162]. Because of the extensive ulceration associated with IBD, it is conceivable that disruption of the mucosal layer in the colon of IBD patients renders them susceptible to invasion of colonic epithelium by *E.coli pks^+^*. The resulting widespread colibactin-associated genome instability could ultimately drive CRC development in such IBD patients. Indeed, in a pathogen-free mouse model of colitis, induction of mucosal barrier disruption increases the invasiveness of *E.coli pks^+^* into the nearby epithelium, where they drive inflammation, DNA damage and cytotoxicity [163], and possibly tumorigenesis. Overall, the data highlight the contribution of the hyper-inflammation and oxidative stress to mutational stress and genome instability in the colonic microenvironment of IBD patients.

### 3.3. Systemic Lupus Erythematosus (SLE)

SLE is a chronic autoimmune disorder affecting roughly 5 million people worldwide. Much like SSc, there is no known cure for SLE, and it predominantly affects women [164]. The etiology of SLE remains obscure, although various diagnosis criteria, ranging from photosensitivity and dermatitis to multisystem disorders such as renal, neurological, hematological, and immune dysfunction are employed in clinical settings [165]. Confoundingly, several immune loci are linked to genetic susceptibility to SLE, most of which belong to the family of interferon regulatory factors (IRFs) (reviewed in [166]). Specifically, IRF5 is a confirmed risk locus for SLE in various ethnic groups and is associated with increased blood levels of interferon IFN-ɑ. Additional variants of immune regulatory factors such as the tumor necrosis factor alpha inducible protein 3 (TNFAIP3), SRC-family kinase BLK, and the transcription factor ETS1, which is a regulator of lymphocyte differentiation, are all associated with elevated risk for SLE development [166]. SLE pathology is intricately connected to B-cell functional abnormalities, such as aberrant cytokine production and increased antigen presentation [167,168].

Several studies have illuminated the extent of DNA damage underlying SLE. It has become evident in recent years that suboptimal detection of genome instability and inefficient DNA repair may have historically obscured SLE disease etiology. Elevated DNA double strand breaks were observed in resting CD4+ T-cells in SLE patients [169]. Similarly, in CD4+ T-cells, the DNA-damage induced gene *GADD45A* was associated with increased demethylation at the *CD11* locus, which is associated with increased autoimmunity in SLE [170]. Prior genome-wide association studies showed that a single nucleotide polymorphism in the DNA base excision repair (BER) gene *POLB* reduced Polβ levels and is correlated with SLE development [171]. A mouse model of SLE that has the Polβ variant Y265C displays classic phenotypes of SLE including nuclear autoantibodies and dermatitis [172]. The likely driver of disease seems to be inefficient BER due to changes in catalytic activity and expression of Pol β, which probably results in elevated genome instability. Other studies have analogously shown defective DNA repair in SLE-derived cells treated with alkylating agents [173], or oxidative damage [174]. Genetics variants of several other base excision repair genes are associated with SLE, such as *XRCC1* [175], DNA glycosylases *OGG1* and *NEIL3* [176,177], and the flap-endonuclease *FEN1* [178]. In addition to DNA repair defects, SLE patients have direct chromatin compaction defects in repair-associated genes and the accumulation of ribonucleotides in DNA [179]. Recently, a newly identified variant of the mismatch repair factor MSH6, which is associated with human SLE, was shown to induce autoantibody production, reduced survival, and pulmonary disease when expressed in mice [180]. B-cell hyperactivity in SLE was shown to be linked to IRF1-mediated activation of the ATR kinase [181]. Further, inhibition of ATR signaling via blocking of the Chk1 effector kinase drastically reduced the production of several inflammatory cytokines in SLE B-cells, providing evidence for a likely role for DNA damage response factors in amplifying hyperactive immune response [181]. Mutations in *TREX1*, which encodes a major mammalian ssDNA exonuclease, leads to DNA damage checkpoint activation and autoimmune phenotypes in mice models [182]) and is associated with SLE and Aicardi–Goutières syndrome (AGS) [183].

The mechanisms that link SLE to DNA damage probably stem from antibody diversification via somatic hypermutation (SHM) and class switch recombination (CSR), nuclear breakdown, and release of DNA into the cytosol, which triggers innate immune response such as TLR9 signaling, along with the cGAS-STING pathway [184,185]. In the absence of effective DNA repair mechanisms, SLE genomes might acquire a large mutational load, which could result in the production of neoantigens. In support of this notion, more than half of all SLE patients share ~20 diagnostic autoantibodies, with some patients harboring up to 180 autoantibodies, which is among the largest autoantibody response among all AID [88]. Such autoimmune responses can trigger neoplasia. Indeed, several meta-analyses of malignancy risks in adult SLE patients have revealed that SLE is associated with an elevated cancer risk compared to the general population. Among cancer types, hematological cancers had the highest risk, followed by lung cancer, non-melanoma skin cancer, and gastrointestinal tumors [89,90,91]. Because SLE is a chronic autoimmune disorder, the pathogenesis of cancer development in SLE patients has been linked to several pathways including oxidative stress, immunomodulatory chemotherapy, autoantibody response, chronic inflammation, and traditional risk factors like smoking [186]. However, it is conceivable that a substantial burden of risk arises from DNA repair defects and/or associated DNA damage, akin to what has been observed in SSc. Curiously, SLE shows an inverse correlation with the risk of developing breast and prostate cancers, suggesting that immune response might act to increase clearance of tumor cells, either via programmed cell death, P53 activation, or suppression of telomerase activity [186]. However, whether these phenomena are correlative or causative towards cancer incidence in SLE are pending investigation.

### 3.4. Multiple Sclerosis

Multiple sclerosis (MS) is a central nervous system disorder affecting more than 2 million people worldwide, and is marked by pronounced muscle weakness, neurodegeneration, and visual impairment, among other symptoms [187]. Immunologically, MS consists of demyelinating plaques that are rich in CD8+ T-lymphocytes, as well as an abundance of Th17 cells that secrete pro-inflammatory cytokines such as IL-6 and TNFɑ, and increase infiltration of the blood–brain barrier by other pro-inflammatory factors [188,189]. PBMCs from MS patients are enriched in the oxidative DNA damage marker 8-oxo-dG [190]. Additionally, SNPs in several DNA repair genes are associated with risk for MS, including nucleotide excision repair (NER) factors XRCC4 and ERCC3, homologous recombination factors RPA1, MRE11, RAD54, and BRCA2 [191]. Genetically, carriers of the MHC allele HLA-DRB1*15:01 have a three-fold elevated risk of developing MS compared to non-carriers, especially within people of northern European descent [192]. Recently, single cell whole-genome sequencing analysis of neurons and oligodendrocytes from MS patients revealed a higher mutation burden in patient cells compared to control cases, and harbored mutational signatures of defective transcriptional-associated DNA repair [193].

### 3.5. Type I Diabetes Mellitus

In Type I diabetes (T1D), a metabolic AID, the Islet of Langerhans are infiltrated by T-lymphocytes, leading to the destruction of a subset of islet β-cells. However, more than a third of β-cells remain intact in T1D, leading researchers to posit mechanisms other than immune infiltration that may promote β-cell dysfunction in T1D. To this end, polymorphisms associated with NER genes *XRCC4*, and non-homologous end joining (NHEJ) factor Ligase IV, were proposed to contribute to β-cell fragility [194]. Further, infiltrated β-cells had extensive DNA damage, as quantified by the intensity of 53BP1 foci formation, a phenomenon that was recapitulated in rodent models of T1D [195]. The rise in DNA damage coincided with the onset of T1D, at which time cells experienced heightened inflammation. It was proposed that this strong DNA damage response in β-cells is driven by a combination of immune dysregulation and metabolic dysregulation, such as increased glycolysis, which likely spikes the production of RONS, thereby enhancing oxidative stress and DNA damaging lesions [195]. Finally, although diabetic patients are at an elevated risk for cancer development, studies aimed at finding the link between T1D and cancer risk have largely yielded mixed results, with marginally elevated risk for gastrointestinal and pancreatic cancer, and lowered risk for breast and prostate cancers [95,96]. Whether or not the association is contingent upon the timeline of cancer detection, co-morbidities like obesity and smoking, or the duration of T1D, warrant detailed investigation.

### 3.6. Rheumatoid Arthritis

Rheumatoid arthritis (RA) is a chronic AID resulting in severe inflammation, joint stiffness, joint damage, and fatigue [196]. Disease development is thought to occur over a period of several years via systematic breakdown of immune tolerance, resulting in autoantibodies to IgA rheumatoid factor (RF), peptidyl arginine deiminase (anti-PAD), and malondialdehyde-acetaldehyde [97,98]. Risk predictors include bacterial and viral infections, environmental factors such as diet and smoking, age, sex, and genetic variation at the HLA locus [197]. Importantly, RA patients are at risk for lymphoma development [99].

To better understand the role of non-immunological factors in the progression of RA, earlier studies focused on how cells lining the inflamed joints can undergo transformation that promotes migration, invasion, and destruction of the extracellular matrix (ECM). In cells obtained from the synovial tissue of RA patients, elevated DNA damage [198] and somatic *TP53* mutations were found to be enriched, a subset of which produced immunoreactive P53 protein [199]. Mitochondrial DNA (mtDNA) isolated from synoviocytes of RA patients had twice as many somatic mutations in the mitochondrially encoded gene *ND1*, compared to those from the non-AID osteoarthritis. In newly diagnosed RA cases, CD8+ T-cells carried a higher proportion of somatic mutations, including mutations in immune-related genes *SLAMF6* and *IRF1* [200]. The overall increased mutation burden in RA likely results from heightened metabolic activity of immune cells in the vicinity of the inflamed tissue. This includes neutrophils and monocytes, causing heightened ROS production and oxidative damage to both nuclear and mitochondrial genomes in the surrounding cells.

### 3.7. The Curious Case of VEXAS

VEXAS (vacuoles, E1 enzyme, X-linked, autoinflammatory, somatic) syndrome is a recently identified late-onset, hematological AID that occurs almost exclusively in men. VEXAS results in severe immunodeficiency arising from progressive bone marrow failure, and results in high morbidity and mortality [201,202]. Unlike most other AID, which have a complex multigenic and environmental pathophysiology, VEXAS uniquely arises monogenically from somatic missense mutations within the *UBA1* gene, which is a master regulator within the ubiquitin pathway, encoding an E1 ubiquitin ligase [203,204]. Sequencing analysis revealed that all three of the initially identified somatic mutations (M41T, M41L, M41V) modified the alternative start codon of the cytoplasmic UBA1 variant, with later studies subsequently identifying additional variants [205,206]. The mechanism(s) underlying systemic inflammation and myeloid restriction of VEXAS mutations are being actively pursued, with a recent study suggesting that *UBA1* mutations can exacerbate apoptosis, and decrease polyubiquitination of proteins in the inflammation pathway [207]. Because multiple steps within the hematopoiesis pathway are regulated via ubiquitylation, mutations in *UBA1* change the relative proportions of nuclear versus cytoplasmic Uba1 variants, and alter the immunological environment within myeloid cells, potentially favoring clonal expansion of mutant cells [208]. Interestingly, UBA1 is involved in DNA damage signaling [209] and regulation of key tumor suppressors such as P53 and C-Myc [210]. As such, mutations resulting in UBA1 mis-localization, loss-of-function, or dysregulation can severely impact overall genome stability and promote mutagenesis and oncogenesis in VEXAS.

## 4. Somatic Mutations and AID—A “Chicken and Egg” Conundrum

Somatic mutations scale with age [2,211,212]. As such, accumulated somatic mutations are functionally associated with the development of several cancers. Therefore, it is reasonable to assume that this also holds true for non-neoplastic age-associated diseases, which include many types of AID as discussed earlier. It has been proposed that not only clonally selected mutations, but random somatic mutations can also severely hamper tissue homeostasis, for example, via increased transcriptional heterogeneity [213,214]. Further, several studies have demonstrated an age-associated increase in somatic mutations in differentiated and post-mitotic cells; single cell sequencing studies revealed a higher mutation load in differentiated hepatocytes compared to liver stem cells [215]. In comparison to naïve lymphocytes, differentiated memory B- and T-cells showed a somatic hypermutation (SHM)-induced increase in mutations [216]. Single cell sequencing of neurons from the pre-frontal cortex and hippocampal regions of brains from individuals ranging from 0.5–80+ years in age found an increase in neurodegeneration and mutations in an age- and disease-related fashion [217]. Overall, these studies strongly suggest that as individuals age, a combination of endogenous and environmental factors shape the mutational landscape across various cells and could pre-dispose individuals to disease [218].

Even so, the causal relationship between somatic mutagenesis and AID is complicated, as several confounding factors can shape disease development, including altered signaling pathways, germline predispositions, and various exogenous interventions. As such, testing the causality has so far been largely restricted to murine models, for example, in SLE and IBD [172,219]. *PIGR* and *NFKBIZ*, which play a key role in the IL-17 pathway, are reported to have a high frequency of somatic mutations in IBD patients. Through knockout experiments in mice, researchers observed that mice lacking a functional IL-17 pathway display symptoms of gut dysbiosis and develop autoimmunity [220,221]. Because *PIGR* and *NFKBIZ* mutant intestinal epithelial cells seem to be under positive selection in IBD patients, it could be hypothesized that this selection is a part of the development of autoimmunity, initially by promoting breakdown of mucosal barriers in the gut, followed by inflammation and selective removal of wildtype crypt cells. In this scenario, somatic mutations would themselves serve as disease drivers.

Nonetheless, establishing a direct relationship between somatic mutations and disease necessitates careful genetic approaches. This could involve combinatorial approaches utilizing gene–gene (GXG) and gene–gene–environment (GXGXE) analyses, wherein the effect of somatic mutations, germline variants, and environmental exposures within a given disease-associated tissue can be simultaneously assessed, much in the vein of genome-wide association studies (GWAS) and quantitative trait loci (QTL) mapping. Model systems can be developed to precisely quantitate age-related accumulation of somatic mutations across different tissues, and/or in response to additional stressors. Lastly, mathematical modeling approaches could be leveraged to understand how somatic evolution contributes to disease-expansion feedback loops, wherein disease drives clonal expansion of mutant clones which then further propagate disease.

## 5. Future Perspectives

The genome-wide somatic mutational burden for a majority of AID has remained largely unexplored. As described in the previous sections, studies with a subset of AID such as SSc and IBD demonstrate that high-throughput sequencing can reveal novel avenues to explore disease status and outcomes. Even so, a key challenge in defining AID risk and outcomes is the accurate identification of somatic mutation loads, the mutational spectra present, and interpretation of their clinical significance. Massively paralleled sequencing technologies are constantly evolving to meet these challenges, with newer technologies offering detection of mutations that occur with low-variant allele frequency (VAF), and with minimal sequencing artifacts.

Somatic mutations are ubiquitous throughout the genomes of normal and diseased cells. The most well-represented mutations with a high-variant allele frequency are readily detected across the genome via bulk sequencing. However, such approaches often fail when analyzing cells that are difficult to propagate in culture, and are ill-suited to identify rare mutations occurring at low frequencies within populations. As an alternate approach, single cell clones can be isolated from bulk populations, which are subsequently sequenced directly [222,223,224], or clonally amplified and then sequenced [225,226,227,228]. These approaches still need considerable computational fine tuning to eliminate sub-clonal mutational calls, false positives, as well as artifactual data from amplification-related errors. On the other hand, one could locally identify mutational processes within the tumor tissue milieu via laser capture microdissection from histological samples [229,230,231,232].

Error-corrected duplex sequencing [233] was developed more than a decade ago to bypass errors arising from amplification bias, sample heterogeneity, and imaging limitations, and has been widely used to identify rare allele variants associated with carcinogenesis and genotoxin exposure [234]. Approaches using nanopore sequencing have circumvented errors of DNA library preparation and sequencing associated with Illumina platforms, utilizing a protein biosensor (nanopore) through which DNA or RNA can translocate and produce electric signals corresponding to the interacting base, predicting the underlying sequence with megabase efficiency (reviewed in [235]). Variants of nanopore sequencing have incorporated the improved base-calling accuracy of duplex sequencing (e.g., [236]) that allows researchers a wider, error-free genome coverage, which could be easily leveraged in the study of mutations in AID. The evolution of novel cell-free DNA (cfDNA) sequencing technologies greatly minimizes the invasiveness of clinical sample collection and processing, while providing accurate, error-corrected SNV detection capabilities. These include an emulsion-PCR based paired plus-minus sequencing (ppmSeq) in plasma-derived cfDNA [237], and flow-based ultra-deep sequencing of cfDNA [238]) from lung and urothelial carcinomas without matched tumor samples. Further application of such approaches in age-, sex-, and tissue-controlled populations of healthy and diseased individuals would illuminate the true landscape of somatic mutagenesis prevalent across various autoimmune disorders and could serve as biomarkers of disease progression.

From a clinical perspective, it is imperative to identify and characterize novel somatic mutations associated with specific AID, as it exposes the repertoire of clinically actionable targets hidden within a patient’s disease microenvironment. Pathogenic germline mutations within DNA repair genes such as *MSH2*, *BRCA1/2,* and *PALB2*, and more recently, the *RAD51* superfamily and are well-studied in the context of multiple cancer types, and subsequently spawned many FDA-approved drug interventions (reviewed in [239]). Somatic mutational data can be similarly utilized as a screening strategy to identify AID patients with an increased likelihood of developing cancers, with high mutational burdens and/or specific driver mutations serving as prognostic biomarkers. Indeed, high-throughput sequencing efforts of the past decade have highlighted several mutations that are targetable via FDA-approved therapeutics. A *BCR-ABL* fusion gene was identified in chronic myeloid leukemia (CML), and is targetable by the tyrosine kinase inhibitor imatinib [240]. Similarly, identification of *EGFR* mutants associated with non-small cell lung cancer (NSCLC) allowed the use of FDA-approved kinase inhibitors afatinib and gefitinib as treatment options [241,242]. Further, melanoma, NSCLC and anaplastic thyroid cancer patients with a *BRAF V600* mutation are approved for treatment with the BRAF inhibitor dabrafenib, either as a monotherapy or in combination with the MEK inhibitor trametinib [243,244,245]. Lastly, because immune cells accumulating a large burden of somatic mutations are expected to be pathogenic, it would allow their pharmacological distinction from normal immune cells, and potentially expand therapeutic options. In line with this notion, rituximab, a monoclonal antibody therapeutic that targets CD20 on B cells in non-Hodgkins’ lymphoma, has been co-opted to selectively target pathogenic B-cells in rheumatoid arthritis in combination with methotrexate [246,247]. Since the etiologies connecting AID and cancer development are largely idiopathic, and because a vast majority of AID genomes remain understudied, somatic mutation analysis could play a key role in illuminating previously unknown targetable pathways and greatly influence disease outcomes.

## 6. Conclusions

There is extensive DNA damage and somatic mutagenesis in AID. While the association of somatic mutations with cancers are extensively documented, recent studies of non-neoplastic diseases have shown that post-zygotic mutation accumulation can have wide-ranging implications in physiological settings. Moreover, the rate of somatic mutation accumulation is widely divergent across species, and varies with age and tissue type, but is on average orders of magnitude more prevalent than germline mutations [225,248,249,250]. Even though most of these changes are phenotypically neutral, selection can confer growth advantages to tissues harboring a subset of mutations, at the expense of overall homeostasis, resulting in disease. In the context of AID that typically are comprised of divergent and seemingly disconnected etiologies, somatic mutations describe the genotoxic profile of immune cells and offer novel clues to disease pathology. Monoallelic mutations often remain dormant, unless the remaining functional copy is inactivated (Knudson’s “two-hit” hypothesis), which elicits a disease, a phenomenon famously postulated as the basis to tumorigenesis [251]. Similarly, immune cells harboring dormant monoallelic variants could potentially become pathogenic through the acquisition of inactivating somatic mutations. Such mutations could functionally disrupt regulatory networks within the cell and confer novel phenotypes on the host cells, allowing for altered self-recognition, clonal hematopoiesis, and stochastic development of autoimmunity. For instance, simple tandem repeats (STRs) are highly mutable, and somatic mutations within protein-coding genes spanning long STRs generate autoantigens, providing a mechanistic link between somatic mutations and autoimmunity [252]. Therefore, identifying somatic mutations, in combination with the patient’s genetic background and environmental exposure profiles, could offer an enhanced view of mechanism of disease onset and progression.

DNA damage and AID can be linked through a variety of different mechanisms (Figure 1). Firstly, unrepaired double strand breaks can lead to micronuclei formation or leakage of nuclear DNA into the cytoplasm, both of which can trigger innate immune responses via cGAS-STING signaling [253]. Secondly, DNA damage can directly activate NF-KB signaling via the activation of the DNA damage checkpoint sensor kinase ATM, inducing inflammation [254]. Thirdly, increased ribonucleotide incorporation in genomic DNA has been linked to the autoimmune syndrome Aicardi–Goutières syndrome (AGS), which shares clinical features with SLE [179]. Lastly, epigenetic changes in response to DNA damage can result in the activation of previously heterochromatinized mobile genetic elements such as LINEs and retrotransposons, which when active can induce DNA and RNA sensing pathway in the cytoplasm and lead to the secretion of interferons [255,256,257]. A combination of these factors can induce inflammatory responses, stochastically promote autoimmunity, and encourage a pro-tumorigenic environment within affected cells. Therefore, identifying the modes of DNA damage and downstream responses to damage sensing across different AID might provide broad insights into common mechanisms operational at the heart of autoimmunity and associated cancers.

## Figures and Tables

**Table 1:** Autoimmune disorders presented in the review, along with their autoantibody status and cancer risk.

Autoimmune Disorder	Affected Tissue/Organ System	Autoantibody Status	Associated Malignancy
Systemic sclerosis (SSc)	Multisystem	CEN, RNAPIII, TOP1 [82]	Lung cancer, gynecological cancers, skin cancers, hematological cancers [83]
Inflammatory bowel disorders (IBD) (including ulcerative colitis (UC) and Crohn’s)	Colonic epithelium	pANCA, anti-pancreas [84]	Colorectal cancer [85,86]
Systemic lupus erythematosus (SLE)	Multisystem	>180, including IL-6, TNF-alpha, CXCL3, VEGF-B, CD44, DNASE1L3 [87,88]	Hematological, non-melanoma skin cancer, lung cancer, gastrointestinal cancer [89,90,91]
Multiple sclerosis (MS)	CNS	Autoantibodies detected against multiple proteins, in astrocytes, neuroglia, oligodendrocytes and blood–brain barrier, including Aquaporins, GLP78, GRP78, glycolipids. Contribution to disease pathology and progression unclear	Slight risk of urogenital cancers [92], DMT-induced risk for some carcinomas including breast and basal cell [92,93]
Type I diabetes mellitus (T1D)	Pancreas	Insulin, GADA, IA-2A, ZnT8A [94]	Slightly elevated risk for pancreatic and GI carcinomas [95,96].
Rheumatoid arthritis (RA)	Musculoskeletal	IgA RF, anti-PAD, MDA/AA [97,98]	Elevated risk for lymphoma [99]
Vacuoles, E1 enzyme, X-linked, autoinflammatory, somatic syndrome (VEXAS)	Hematological	Autoantibody negative	Increased risk for hematological cancers [100]

**Table 2:** DNA damage and mutagenesis reported in autoimmune disorders. ^●^ CIN—centromere instability, SSB—single strand breaks, DSB—double strand breaks. ^†^ PBMCs—peripheral blood mononucleocytes; **^‡^** SBS—single base substitutions, DBS—doublet base substitutions, MBS—multibase substitutions, SV—structural variations, CNV—copy number variation, INDEL—insertion-deletion, SNV—single nucleotide variation; * WES—whole exome sequencing, WGS—whole genome sequencing, IF—indirect immunofluorescence.

Disease	DNA Damage ^●^	Cell/Sample Type ^†^	Somatic Mutation Type ^‡^	Method *	Mutation Spectra	Refs.
SSc (Systemic sclerosis)	CIN, MN	Skin fibroblasts	NR	qPCR, IF	-------	[101]
	8-oxoG lesions	Urine	NR	ELISA	-------	[102]
	Telomere attrition	Leukocytes	NR	Southern blot	-------	[103]
	SSB, DSB	PBMCs	NR	Comet assay	-------	[104]
	Mutations	Skin fibroblasts	SBS	WES	SBS5, SBS40, *KRAS*, *TP53*, *PIK3CA* mutations	[105]
	Mutations	Lung fibroblasts	SBS, DBS, MBS, CNV, SV	WGS	SBS2, SBS13 (*APOBEC*), SBS 93, nTw → N (*POLH*), wrC → T (*AICDA*), mutations in cancer drivers (*NF1*, *SEC31*), inflammation and immune response (*CTNNA3*, *BCOR*), DNA damage response (*CGAS*)	[55]
Inflammatory Bowel Disease (IBD)	MN, nucleoplasmic bridges, oxidative damage	Lymphocytes			---------	[106]
	Mutations	Colon epithelia	SBS	WES	*RHO*, *RAC*, *IL16*, *NRG1*, *TP53*, *APC*, *IDH1*	[107]
	Mutations	Colon epithelia	SBS, INDELS	WGS	ID1,2 (replication slippage), SBS2,13 (APOBEC), SBS18 (ROS), SBS1,5 (clock-like), clonally expanded driver mutations (*KRAS*, *TP53*, *BRSF*, *ATM*, and *SOX9*)	[108]
	Mutations	Colon organoids	SBS	WES	*NFKBIZ TRAF3IP2* mutations (+selection in non-cancer UC)	[109]
	Mutations	Non-cancer IBD patients	SBS	WGS, WES	aTn → aCn (temozolamide), gCn → gAn (acetaldehyde), tCw → tGw (APOBEC), nCg → N (clock like)	[110]
SLE	Chromatin defects, oxidative damage, rNTP incorporation in DNA	Lymphocytes, PBMCs	NR	IF, SS	---------	[111,112,113]
MS	8-oxoG lesions	PBMCs	----	Comet assay	---------	[114]
	Mutations	Neurons, oligodendrocytes	SNVs	WGS	SBS44 (MMR), SBS30 (BER), SBS5 (age-associated), SBS19, C → T TSB	[115]
T1D	53BP1 foci	Islet beta cells	----	IF	---------	[116]
RA	DNA damage, mutations	Synoviocytes	SNVs	RMD, SS	*TP53* hotspot mutations	[117]
	Mutations	Synoviocytes	SNVs	ES	Mitochondrial DNA mutations (*ND1*)	[118]
	Mutations	CD8+ T-cells	SNVs	ES	Immune gene mutations (*SLAMF6*, *IRF1*)	[118]
VEXAS	Mutations	Myeloid-derived cells	SNVs	SS	*UBA1* mutations	[119,120,121]

## Data Availability

No new data were created or analyzed in this study. Data sharing is not applicable to this article.

## Abbreviations

The following abbreviations are used in this manuscript:

4-HNE	4-hydroxynonenal
AGS	Aicardi–Goutières syndrome
AID	Autoimmune disease
ASCS	Australian Scleroderma Cohort Study
BER	Base excision repair
cfDNA	Cell-free DNA
CNV	Copy number variation
COPD	Chronic obstructive pulmonary disease
COSMIC	Catalog of somatic mutations in cancers
CRC	Colorectal cancer
DBS	Double base substitutions
DDR	DNA damage response
DSB	Double strand breaks
DSS	Dextran sodium sulfate
ecDNA	Extrachromosomal DNA
ECM	Extracellular matrix
HLA	Human leukocyte antigen
HNPCC	Hereditary non-polyposis colorectal cancer
IBD	Inflammatory bowel disorders
ILD	Interstitial lung disease
INDEL	Insertions-deletions
IRF	Interferon regulatory factor
MDA	Malondialdehyde
MDSC	Myeloid derived suppressor cells
MMR	Mismatch repair
MS	Multiple sclerosis
NER	Nucleotide excision repair
NHEJ	Non-homologous end joining
PBMC	Peripheral blood mononucleocytes
PDGF	Platelet-derived growth factor
PCD	Programmed cell death
RA	Rheumatoid arthritis
RONS	Reactive oxygen and nitrogen species
ROS	Reactive oxygen species
SARD	Severe autoimmune rheumatic disease
SBS	Single base substitution
SHM	Somatic hypermutation
SLE	Systemic lupus erythematosus
SNP	Single nucleotide polymorphism
SNV	Single nucleotide variant
SSB	Single strand breaks
STM	Simple tandem repeats
T1D	Type I diabetes mellitus
TIL	Tumor-infiltrating lymphocytes
UC	Ulcerative colitis
VAF	Variant allele frequency
VEXAS	Vacuoles, E1 enzyme, X-linked, autoinflammatory, somatic syndrome
WES	Whole exome sequencing
WGS	Whole genome sequencing
